# The superiority of high‐power short‐duration radiofrequency catheter ablation strategy for atrial fibrillation treatment: A systematic review and meta‐analysis study

**DOI:** 10.1002/joa3.12590

**Published:** 2021-07-02

**Authors:** Yoga Waranugraha, Ardian Rizal, Achmad J. Firdaus, Fransiska A. Sihotang, Akita R. Akbar, Defyna D. Lestari, Muhammad Firdaus, Akhmad I. Nurudinulloh

**Affiliations:** ^1^ Department of Cardiology and Vascular Medicine Faculty of Medicine Universitas Brawijaya, Dr. Saiful Anwar General Hospital Malang Indonesia; ^2^ Brawijaya Cardiovascular Research Center Universitas Brawijaya Malang Indonesia

**Keywords:** atrial fibrillation, catheter ablation, high‐power short duration, low‐power long‐duration, meta‐analysis

## Abstract

**Background:**

Radiofrequency catheter ablation (RFCA) using the high‐power short duration (HPSD) results in better ablation lesion formation in the swine model. This systematic review and meta‐analysis purposed to investigate the safety and efficacy profile between HPSD and low‐power long‐duration (LPLD) ablation strategies to treat atrial fibrillation (AF) patients.

**Methods:**

We completed the literature review after identifying the relevant articles comparing HPSD and LPLD ablation methods for AF recorded in ClinicalTrials.com, CENTRAL, PubMed, and ScienceDirect until February 2021. The overall effects were calculated using pooled risk ratio (RR) and mean difference (MD) for categorical and continuous data, respectively. We also estimated the 95% confidence interval (CI).

**Results:**

The HPSD strategy took shorter procedure time (MD = −33.75 min; 95% CI = −44.54 to −22.97; *P* < .01), fluoroscopy time (MD = −5.73 min; 95% CI = −8.77 to −2.70; *P* < .001), and ablation time (MD = −17.71; 95% CI = −21.02 to −14.41) than LPLD strategy. The HPSD RFCA was correlated with lower risk of esophageal thermal injury (RR = 0.75; 95% CI = 0.59 to 0.94; *P* = .02). The HPSD method resulted in higher first‐pass pulmonary vein isolation (PVI) (RR = 1.36; 95% CI = 1.13 to 1.64; *P* < .01), lower PV reconnection (RR = 0.47; 95% CI = 0.34 to 0.64; *P* < .01), and lower recurrent AF (RR = 0.72; 95% CI = 0.54 to 0.96; *P* = .02) than LPLD strategy.

**Conclusion:**

HPSD RFCA was superior to the conventional LPLD RFCA in terms of safety and efficacy in treating AF patients.

## Graphical abstract

Radiofrequency catheter ablation (RFCA) using the high‐power short‐duration (HPSD) results in better ablation lesion formation in the swine model. This systematic review and meta‐analysis purposed to investigate the safety and efficacy profile between HPSD and low‐power long‐duration ablation strategies to treat atrial fibrillation (AF) patients. Our systematic review and meta‐analysis study revealed that the HPSD RFCA was a safe, effective, and efficient procedure to treat AF.

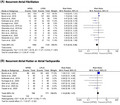

## 1 INTRODUCTION

Compared with the optimal medical treatment (OMT), catheter ablation results in better atrial fibrillation (AF) outcomes.^1^, ^2^ Catheter ablation for pulmonary vein isolation (PVI) is recommended by the current guideline to restore the sinus rhythm in paroxysmal AF or persistent AF.^3^ The sinus rhythm is successfully maintained in 60.8%‐71% of AF patients following the catheter ablation procedure.^4^ The complete PVI can be achieved through permanent, continuous, and transmural tissue damage using radiofrequency catheter ablation (RFCA).^5^ However, several complications, such as pericardial effusion/tamponade, esophageal injury, vascular access complication, or pulmonary vein (PV) stenosis, can occur during RFCA procedure.^6^, ^7^ Inappropriate energy delivery might be the possible cause of the procedural complications and failure in sinus rhythm preservation.

RFCA induces thermal injury through resistive and conductive heating. The equilibrium between power and duration of radiofrequency (RF) delivery during resistive and conductive heating is a critical determinant for lesion generation. The resistive heating directly leads to permanent myocardial tissue damage with necrosis, whereas conductive heating spreads to the deeper tissue layers, leading to reversible damage in myocardial tissue.^5^, ^8^, ^9^, ^10^, ^11^ In daily clinical practice, the low‐power long‐duration (LPLD) ablation strategy is more commonly used.^12^ That conventional method is correlated with longer RF application time, longer conduction heating, and deeper tissue heating.^5^, ^8^, ^9^, ^10^, ^11^ So, the risk of complications is predicted to be higher. The new approach called the high‐power short‐duration (HPSD) ablation strategy might be used to overcome those limitations.^5^, ^9^, ^13^ In silico and animal studies demonstrated that catheter ablation using the HPSD approach resulted in shorter ablation time, better linear continuity, better lesion uniformity, and better lesion transmurality.^5^, ^9^ However, the safety and efficacy profile of HPSD and LPLD ablation strategies in humans is still unclear. Therefore, we conducted a systematic review and meta‐analysis to investigate the safety and efficacy profile between HPSD and LPLD ablation strategies for AF treatment.

## 2 METHODS

This systematic review and meta‐analysis were conducted based on preferred reporting items for systematic reviews and meta‐analyses (PRISMA).^14^

### 2.1 Literature search

We searched for and identified the relevant studies comparing HPSD and LPLD ablation strategies for AF patients from the electronic scientific databases such as ClinicalTrials.com, CENTRAL, PubMed, and ScienceDirect. We applied the following keywords during the literature searching process: (“catheter ablation” OR “radiofrequency ablation” OR “RF ablation” OR “RFA” OR “radiofrequency catheter ablation” OR “RFCA” OR “ablation”) AND (“high‐power short‐duration” OR “HPSD”) AND (“low‐power long‐duration” OR “LPLD”) AND (“atrial fibrillation” OR “AFib” OR “AF”). We completed the literature searching process in February 2021. Three investigators conducted the literature search.

### 2.2 Eligibility criteria

We included the studies with the following criteria: (i) original research articles comparing HPSD and LPLD RFCA strategies for AF, (ii) the aim of RFCA was for rhythm control, (iii) article written in English, (iv) availability of the data about power and duration during RF delivery, and (v) availability of the detailed information about the treatment, procedural aspects, safety outcomes, and efficacy outcomes. We also excluded articles with the following criteria: (i) duplications, (ii) the full‐text manuscript unavailability, (iii) the article used the data from similar studies, (iv) incomparable treatment group and control group, (v) ablation index (AI) guided catheter ablation, and (vi) outcomes of interest were not reported. The study selection process was performed by three investigators.

### 2.3 Exposure and outcomes

The exposure was the RFCA method. Patients were classified into the “HPSD group” and “LPLD group.” HPSD was defined as the catheter ablation performed using the highest Power ≥40 W and duration ≤10 seconds in any ablation or less than duration in the LPLD group. In comparison, LPLD was defined as the catheter ablation performed using the highest power <40 W and duration ≥10 seconds in any ablation or longer than duration in the HPSD group. The outcomes measured included: procedural aspects (procedure time, fluoroscopy time, and ablation time), safety outcomes (esophageal thermal injury [ETI], pericardial effusion or cardiac tamponade, and phrenic nerve paralysis [PNP]), and efficacy outcomes (first‐pass PVI, pulmonary vein reconnection [PVR], recurrent AF, and recurrent atrial flutter [AFL] or atrial tachycardia [AT]).

### 2.4 Study quality assessment and data extraction

All eligible randomized controlled trials (RCTs) and cohort studies comparing HPSD and LPLD ablation strategies for AF patients were involved in this study. The quality assessment of RCTs was performed using the modified Jadad scale, which ranged from 0 to 8.^15^ A good‐quality RCT is defined as an RCT with a modified Jadad score ranged from 4 to 8.^16^ For cohort studies, study quality assessment was completed using the Newcastle‐Ottawa scale (NOS). According to the NOS, a good quality cohort study was defined as a study with 3‐4 stars in the selection area, 1‐2 stars in the comparability area, and 2‐3 stars in the outcome area.^17^ To minimize the risk of bias in this systematic review and meta‐analysis, we only involved high‐quality studies. Two investigators conducted the study quality assessment. The disagreement between both investigators was resolved through discussion and the second opinion of the third investigator.

The essential data about: (i) the name of the first author; (ii) publication date; (iii) design of the study; (iv) center involved; (v) number of patients; (vi) AF type; (vii) ablation strategy; (viii) HPSD ablation criteria; (ix) LPLD ablation criteria; (x) length follow‐up period; (xi) arrhythmia detection method; (xii) demographic data (sex and age); (xiii) CHA_2_DS_2_‐VASc score; (xiv) comorbid diseases such as hypertension, diabetes mellitus (DM), heart failure (HF), coronary artery disease (CAD), stroke, or transient ischemic attack (TIA); (xv) echocardiographic parameters such as left atrial diameter (LAD), left atrial volume index (LAVI), or left ventricular ejection fraction (LVEF); (xvi) procedural aspects (procedure time, fluoroscopy time, or ablation time); (xvii) safety outcomes (ETI, pericardial effusion, cardiac tamponade, or PNP); and (xviii) efficacy outcomes (first‐pass PVI, pulmonary vein reconnection (PVR), recurrent AF, recurrent AFL, and recurrent AT) were obtained from each article. Three investigators performed the data extraction process. We reported the categorical data and continuous data using number (percentage) and mean ± standard deviation (SD), respectively. For continuous data, we also quantified mean ± SD from the median and interquartile range (IQR).^18^, ^19^, ^20^

### 2.5 Statistical analysis

The statistical analysis was completed based on the standard guideline.^21^ Assessment of heterogeneity and potential publication bias was conducted before the conclusion determination. The *Q*‐test was used to assess the heterogeneity. We used a cut‐off point of 0.1 for *P* for heterogeneity. We used the random‐effect analysis model in the presence of heterogeneity (*P* < .1). On the other hand, in the absence of heterogeneity (*P* ≥ .1), we used the fixed‐effect analysis model.^22^ We applied the combination of Begg’s and Egger’s tests to assess the presence of publication bias. The *P*‐value of Begg’s test and/or Egger’s test <.05 indicated the presence of publication bias.^23^ The pooled risk ratio (RR) and 95% CI for categorical data were calculated using the Mantel–Haenszel statistical method. The pooled mean difference (MD) and 95% CI for continuous data were determined using the inverse variance statistical method. A *P*‐value of <.05 was considered significant statistically.^24^ Both Review Manager Version 5.3 (Cochrane, Copenhagen, Denmark) and Comprehensive Meta‐Analysis version 3.0 (CMA, New Jersey, USA) were used in the data analysis process. Two investigators conducted the statistical analysis.

## 3 RESULTS

### 3.1 Eligible studies

**FIGURE 1:**
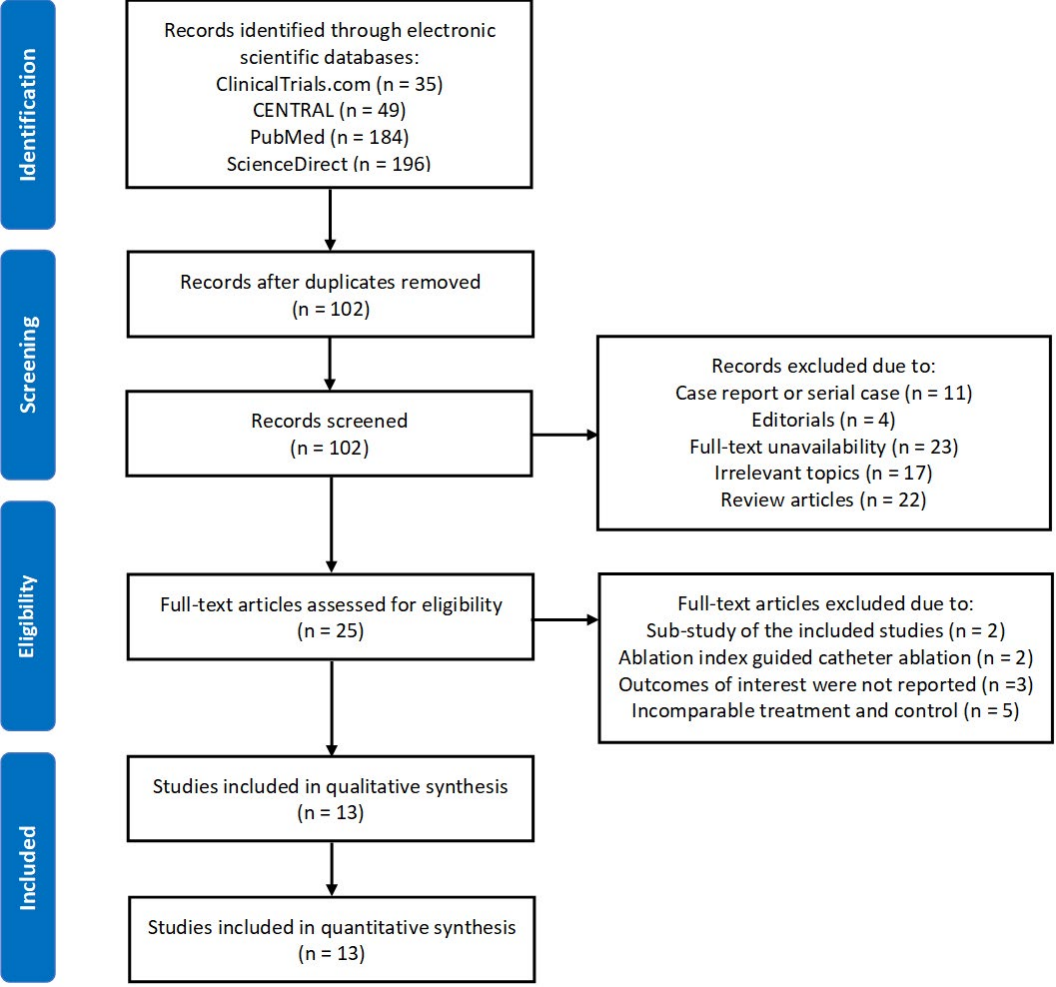
Flow diagram showing study selection process

In the beginning, we successfully obtained a total of 464 records from ClinicalTrials.com (n = 35), CENTRAL (n = 49), PubMed (n = 184), and ScienceDirect (n = 196). After duplicates removal, we still had 102 records. In the next step, 77 records were removed because of this several reasons: (i) case reports or serial cases (n = 11), (ii) editorials (n = 4), full‐text unavailability (n = 23), irrelevant topics (n = 17), and review articles (n = 22). A total of 25 articles were processed in the last step of the eligibility assessment. In this step, we excluded 12 studies because of: (i) substudy of the included studies (n = 2), (ii) AI guided catheter ablation (n = 2), (iii) outcomes of interest were not reported (n = 3), and (iv) incomparable treatment and control. In the end, we had 13 studies to be included in qualitative and quantitative data synthesis.^25^, ^26^, ^27^, ^28^, ^29^, ^30^, ^31^, ^32^, ^33^, ^34^, ^35^, ^36^, ^37^ Figure 1 represents the study selection process.

### 3.2 Baseline characteristics

In minimizing the risk of bias, we only included high‐quality studies. We had 2 RCTs and 11 cohort studies in this systematic review and meta‐analysis study.^25^, ^26^, ^27^, ^28^, ^29^, ^30^, ^31^, ^32^, ^33^, ^34^, ^35^, ^36^, ^37^ Most of them were single‐center studies.^25^, ^26^, ^27^, ^28^, ^29^, ^30^, ^31^, ^34^, ^35^, ^36^, ^37^ Four studies only included paroxysmal AF patients, while 9 studies include paroxysmal AF and nonparoxysmal AF patients.^25^, ^26^, ^27^, ^30^, ^31^, ^33^, ^34^, ^36^, ^37^ Only two studies used PVI only ablation,^29^, ^32^ whereas other studies used the combination of linear ablation, box isolation, superior vena cava isolation, cavotricuspid isthmus ablation, and/or another non‐pulmonary vein (non‐PV) foci ablation in addition to PVI.^25^, ^26^, ^27^, ^28^, ^30^, ^31^, ^33^, ^34^, ^35^, ^36^, ^37^ The follow‐up period duration of the study from Castrejón‐Castrejón et al. was 3 days because they assessed the safety and feasibility of the HPSD ablation strategy.^27^ However, the follow‐up period of other studies varied from 6 months to 2.5 years.^25^, ^26^, ^28^, ^29^, ^30^, ^31^, ^32^, ^33^, ^34^, ^35^, ^36^, ^37^ Arrhythmia detection methods included 12‐lead electrocardiography (ECG), Holter monitor, portable ECG monitor, or telemetry ECG recorder.^25^, ^26^, ^28^, ^29^, ^30^, ^31^, ^32^, ^33^, ^34^, ^35^, ^36^, ^37^ Table 1 summarizes the baseline characteristics of the involved studies.

**TABLE 1:** Baseline characteristics of the included studies

Study	Design	Patients	AF type	Ablation strategy	HPSD	LPLD	Length of the follow‐up period	Arrhythmia detection method
Baher et al., 2018^25^	SC‐RC	687	PAF NPAF	PVI LA PWD	CF or non‐CF sensing Power 50 W Duration 5 s Temperature ≤50°C CF = 10‐20 g	CF or non‐CF sensing Power ≤35 Duration 10‐30 s Temperature ≤50°C CF 10‐20 g	2.5 y	ECG Event monitor
Bunch et al., 2019^26^	SC‐RC	804	PAF NPAF	PVI LA	CF or non‐CF sensing Power 50 W Duration 2‐3 s (PW); 5‐15 s (AW) CF 5‐20 g	CF or non‐CF sensing Power 30 W Duration 3‐10 s (PW); 10‐20 s (AW) CF 5‐20 g	3 y	ECG 24‐Holter monitor Event monitor
Castrejón‐ Castrejón et al., 2020^27^	SC‐PC	95	PAF NPAF	PVI CTIA LA SVCI	CF sensing Power 60 W (a); 50 W (b) Duration 7‐10 s Temperature ≤45°C	Non‐CF sensing Power 30 W Duration 30 s Temperature ≤45°C	3 d	NA
Ejima et al., 2020^28^	SC‐PC	120	PAF	PVI LA SVCI	CF sensing Power 50 W Duration 3‐5 s Temperature ≤42°C CF 5‐20 g	CF sensing Power 25‐40 W Duration 5‐10 s Temperature ≤42°C CF 10‐20 g	20.7 ± 2 mo	ECG 24‐h Holter monitor Portable ECG monitor
Kottmaier et al., 2019^29^	SC‐PC	197	PAF	PVI	Power 70 W Duration 5‐7 s	Power 30‐40 W Duration 20‐40 s	12 mo	ECG 7‐d Holter monitor
Kumagai et al., 2020^30^	SC‐PC	160	PAF NPAF	PVI CTIA LA SVCI	CF sensing Power 50 W Duration 5 s Temperature ≤42°C	CF sensing Power 20‐40 W Duration 30 s Temperature ≤42°C	14.3 ± 3 mo	ECG 24‐h/7‐d Holter monitor Telemetry ECG recorder
Okamatsu et al., 2019^31^	SC‐PC	60	PAF NPAF	PVI BOXI CTIA LA SVCI	CF sensing Power 30‐50 W Duration 7‐10 s CF 10‐20 g	CF sensing Power 30‐40 W (a); 20‐30 W (b) Duration 7–12 s (a); 11‐18 s (b) CF 10‐20 g	6 mo	ECG 24‐h Holter monitor Portable ECG monitor
Pamburn et al., 2019^32^	MC‐PC	100	PAF	PVI	CF sensing Duration 8.5 ± 0.8 s Power 40‐50 W	CF sensing Duration 15.7 ± 2.3 s Power 25‐30 W	12 mo	ECG 24‐h/10‐d Holter monitor
Shin et al., 2020^33^	MC‐RCT	150	PAF NPAF	PVI BOXI LA	CF sensing Power 50 W Duration 10 s Temperature ≤40°C	CF sensing Power = 40 W (a); 30 W (b) Duration = 20 s (a); 40 s (b) Temperature ≤ 40°C	12 mo	ECG 24‐h Holter monitor
Vassallo et al., 2020^34^	SC‐RC	144	PAF NPAF	PVI CTIA	CF sensing Power 45 W (PW); 50 W (AW) Duration 6 s CF 5‐10;10‐20 g	CF‐sensing Power 20 W (PW); 30 W (AW) Duration 30 s CF 10‐30 g	12 mo	ECG 24‐h Holter monitor
Wielandts et al., 2021^35^	SC‐RCT	96	PAF	PVI CTIA	CF‐sensing Power 45 W Duration 13 (11‐14) s (PW); 26 (23‐28) s (other)	CF sensing Power 35 W Duration 17 (16‐19) s (PW); 37 (32‐42) s (other)	6 mo	ECG 24‐h Holter monitor
Yavin et al., 2020^36^	SC‐PC	224	PAF NPAF	PVI LA CTIA	CF sensing Power 45‐50 W Duration 8 s (PW); 15 s (other) Temperature ≤39°C	CF sensing Power 20 W (PW); 30‐40 W (other) Duration 20 s (PW); 30 s (other) Temperature ≤39°C	HPSD 1.2 (0.2‐2.9) y LPLD 1.9 (0.3‐3.7) y	ECG 14‐d Holter monitor Patient triggered Holter monitor
Yazaki et al., 2020^37^	SC‐RC	64	PAF NPAF	PVI LA Non‐PV foci ablation	CF sensing Power 50 W Duration 5‐10 s Temperature ≤42°C	CF sensing Power 20‐25 W (PW); 25‐40 W (other) Duration 15 s (PW); 30 s (other) Temperature ≤42°C	10 (4‐12) mo	ECG 24‐h Holter monitor Portable ECG monitor

Abbreviations

AF, atrial fibrillation; AW, anterior wall; BOXI, box isolation; CF, contact force; CTIA, cavotricuspid isthmus ablation; ECG, electrocardiogram; HPSD, high‐power short duration; LA, linear ablation; LPLD, low‐power long duration; MC, multicenter; NA, not available; NPAF, nonparoxysmal AF; PAF, paroxysmal AF; PC, prospective cohort; PVI, pulmonary vein isolation; PW, posterior wall; PWD, posterior wall debulking; RC, retrospective cohort; RCT, randomized controlled trial; SC, single center; SVCI, superior vena cava isolation.

A total of 2901 patients, including 1644 patients in HPSD group and 1257 patients in LPLD group, were included in the data analysis. The mean age of the included patients varied from 57.3 to 68.3 years old. Male patients contributed in 55‐84% of all included patients.^25^, ^26^, ^27^, ^28^, ^29^, ^30^, ^31^, ^32^, ^33^, ^34^, ^35^, ^36^, ^37^ The mean CHA_2_DS_2_‐VASc score ranged from 1.8 to 2.9.^25^, ^28^, ^33^, ^36^ The prevalence of comorbid diseases, such as hypertension, diabetes mellitus, heart failure, CAD, and stroke/TIA, were 24%‐89.1%,^25^, ^26^, ^28^, ^29^, ^31^, ^32^, ^33^, ^34^, ^36^ 5%‐31.3%,^25^, ^26^, ^28^, ^31^, ^32^, ^33^, ^34^, ^36^ 0%‐46.8%,^25^, ^26^, ^31^, ^33^ 9%‐22.6%,^25^, ^29^ and 0%‐15%,^25^, ^28^, ^29^, ^31^, ^32^, ^33^, ^34^ respectively. The mean LAD ranged from 39 to 47.1 mm.^30^, ^31^, ^33^, ^35^, ^36^ On the other hand, the mean LAVI varied from 34.3 to 41 mL/m^2^.^28^, ^37^ Most patients had good left ventricular (LV) systolic function with mean LVEF of 54.6%‐62.5%.^26^, ^27^, ^28^, ^29^, ^30^, ^32^, ^33^, ^36^, ^37^ Summary of the baseline characteristics of the patients are presented in Table 2.

**TABLE 2:** Baseline characteristics of the included patients

Study	Arm	Patients	Male	Age (y)	PAF	CHA_2_DS_2_‐VASc	HT	DM	HF	CAD	Stroke/TIA	LAD (mm)	LAVI (mL/m^2^)	LVEF (%)
Baher et al., 2018^25^	HPSD	574	385 (67.1)	69 ± 11.8	276 (46.8)	2.9 ± 1.7	369 (64.2)	112 (19.5)	89 (15.5)	130 (22.6)	81 (14.1)	NA	NA	NA
	LPLD	113	67 (59.3)	68.3 ± 11.6	80 (70.8)	2.5 ± 1.6	68 (60.1)	18 (18.5)	15 (13.2)	20 (17.7)	7 (6.2)	NA	NA	NA
Bunch et al., 2019^26^	HPSD	402	253 (62.9)	67.1 ± 10.5	202 (50.2)	NA	358 (89.1)	126 (31.3)	190 (47.3)	NA	NA	NA	NA	54.6 ± 12.1
	LPLD	402	262 (65.2)	66.4 ± 12.2	190 (47.3)	NA	348 (86.6)	121 (30.1)	188 (46.8)	NA	NA	NA	NA	54.7 ± 12.8
Castrejón‐Castrejón et al., 2020^27^	HPSD (a)	30	32 (67)	61 ± 10	31 (65)	NA	NA	NA	NA	NA	NA	NA	NA	57 ± 9
	HPSD (b)	18												
	LPLD	47	28 (60)	61 ± 10	30 (64)	NA	NA	NA	NA	NA	NA	NA	NA	56 ± 11
Ejima et al., 2020^28^	HPSD	60	44 (73)	63 ± 11.3	60 (100)	1.8 ± 1.4	29 (48)	10 (17)	NA	NA	6 (10)	NA	34.3 ± 10.3	57.7 ± 3.9
	LPLD	60	42 (70)	66.7 ± 8.9	60 (100)	2.2 ± 1.4	30 (50)	12 (20)	NA	NA	7 (12)	NA	36.1 ± 8.7	57.4 ± 6.3
Kottmaier et al., 2019^29^	HPSD	97	57 (58.8)	60.8 ± 13.9	97 (100)	NA	56 (57.7)	NA	NA	13 (13.4)	6 (6.2)	NA	NA	57 ± 5
	LPLD	100	60 (60)	60.8 ± 10.5	100 (100)	NA	58 (58)	NA	NA	9 (9)	7 (7)	NA	NA	55 ± 9
Kumagai et al., 2020^30^	HPSD	80	60 (75)	63 ± 9.1	30 (37.5)	NA	NA	NA	NA	NA	NA	41.6 ± 5.1	NA	62.5 ± 7.7
	LPLD	80	66 (82.5)	63.1 ± 9.1	24 (30)	NA	NA	NA	NA	NA	NA	43.3 ± 6.4	NA	62.2 ± 7.2
Okamatsu et al., 2019^31^	HPSD	20	13 (65)	65 ± 10	13 (65)	2 (1 ‐ 3)	10 (50)	5 (25)	0 (0)	NA	0 (0)	40 ± 6	NA	65 (60 ‐ 71)
	LPLD (a)	20	11 (55)	64 ± 8	15 (75)	2 (1 ‐ 3)	8 (40)	2 (10)	2 (10)	NA	3 (15)	40 ± 5	NA	64 (59 ‐ 71)
	LPLD (b)	20	15 (75)	68 ± 8	16 (80)	2 (1 ‐2)	10 (50)	1 (5)	1 (5)	NA	0 (0)	39 ± 6	NA	64 (60 ‐ 67)
Pamburn et al., 2019^32^	HPSD	50	35 (70)	65 ± 8.2	50 (100)	NA	14 (28)	3 (6)	NA	NA	3 (6)	NA	NA	61.7 ± 5.6
	LPLD	50	30 (60)	62.5 ± 10.6	50 (100)	NA	12 (24)	3 (6)	NA	NA	3 (6)	NA	NA	61.1 ± 4.4
Shin et al., 2020^33^	HPSD	50	39 (78)	58.5 ± 7.9	25 (50)	1.6 ± 1.5	24 (48)	8 (16)	13 (26)	NA	7 (14)	39.9 ± 4.6	NA	55.7 ± 11.4
	LPLD (a)	50	42 (84)	57.3 ± 10.8	23 (46)	1.7 ± 1.3	32 (64)	13 (26)	8 (16)	NA	5 (10)	41.5 ± 6.3	NA	57.6 ± 12
	LPLD (b)	50	33 (66)	58.7 ± 11.1	24 (48)	1.7 ± 1.6	22 (44)	8 (16)	5 (10)	NA	6 (12)	40.7 ± 6.5	NA	58.9 ± 8.3
Vassallo et al., 2020^34^	HPSD	71	50 (70.4)	59.7 (med)	39 (54.9)	3 (0 ‐ 8)	52 (73.2)	20 (28.2)	NA	NA	10 (14.1)	NA	NA	NA
	LPLD	73	50 (68.5)	60.7 (med)	52 (71.2)	2 (0 ‐ 7)	53 (72.6)	14 (19.2)	NA	NA	8 (10.1)	NA	NA	NA
Wielandts et al., 2021^35^	HPSD	48	32 (67)	64 ± 11	48 (100)	1 (0 ‐ 3)	NA	NA	NA	NA	NA	39 ± 7	NA	NA
	LPLD	48	33 (69)	64 ± 11	48 (100)	1 (0 ‐ 3)	NA	NA	NA	NA	NA	40 ± 7	NA	NA
Yavin et al., 2020^36^	HPSD	112	71 (63.3)	62.3 ± 5.2	76 (67.8)	2.4 ± 1.3	70 (62.5)	11 (9.8)	NA	NA	NA	44.2 ± 4.7	NA	60.3 ± 6.1
	LPLD	112	79 (70.5)	64.8 ± 7.2	67 (59.8)	2.6 ± 1.4	76 (67.8)	7 (6.2)	NA	NA	NA	47.1 ± 5.1	NA	57.8 ± 5.4
Yazaki et al., 2020^37^	HPSD	32	27 (84)	61 ± 11	22 (89)	NA	NA	NA	NA	NA	NA	NA	40 ± 13	55 ± 7
	LPLD	32	20 (63)	66 ± 11	22 (89)	NA	NA	NA	NA	NA	NA	NA	41 ± 14	56 ± 7

Abbreviations

CAD, coronary artery disease; DM, diabetes mellitus; HF, heart failure; HPSD, high‐power short duration; HT, hypertension; LAD, left anterior descending; LAVI, left atrial volume index; LPLD, low‐power long duration; LVEF, left ventricular ejection fraction; NA, not available; PAF, paroxysmal atrial fibrillation; TIA, transient ischemic attack.

### 3.3 Study quality and publication bias

Based on the assessment using the modified Jadad scale for RCTs (Table S1) and NOS for cohort studies (Table S2), we only included good‐quality studies in our analysis. It was our effort to minimize the risk of bias. Moreover, we did not find any publication bias because no *P*‐values of <.05 were obtained from Begg’s and Egger’s tests (Tables 3 and 4).

**TABLE 3:** Summary of procedural parameters

Parameters	Number of studies	HPSD, n	LPSD, n	Model	MD, min	95% CI, min	*P*‐value of heterogeneity	*P*‐value of Begg’s test	*P*‐value of Egger’s test	*P*
Procedure time	12	1491	1079	Random	−33.75	−44.54 to −22.97	<.01	.45	.16	<.01
Fluoroscopy time	11	917	966	Random	−5.73	−8.77 to −2.70	<.01	.06	.37	<.01
Ablation time	9	759	553	Random	−17.71	−21.02 to −14.41	<.01	.92	.87	<.01

Abbreviations

CI, confidence interval; HPSD, high‐power short duration; LPLD, low‐power long duration; MD, mean difference.

**TABLE 4:** Summary of safety and efficacy outcomes

Parameters	Number of studies	HPSD			LPLD		Model	RR	95% CI	*P*‐value of heterogeneity	*P*‐value of Begg’s test	*P*‐value of Egger’s test	*P*
		Event, n	Total, n	Event, n		Total, n							
ETI	4	207	670	75		255	Fixed	0.75	0.59 to 0.94	.26	.31	.42	.02
PE or cardiac tamponade	5	3	306	10		355	Fixed	0.55	0.19 to 1.62	.74	.46	.09	.28
PNP	3	2	204	1		204	Fixed	1.40	0.28 to 7.02	.54	.29	.44	.68
First‐pass PVI	8	470	553	393		639	Random	1.36	1.13 to 1.64	<.01	.35	.22	<.01
PVR	8	52	979	121		1069	Fixed	0.47	0.34 to 0.64	.46	.27	.13	<.01
Recurrent AF	12	433	1582	280		1125	Random	0.72	0.54 to 0.96	.01	.78	.13	.02
Recurrent AFL or AT	7	104	769	91		771	Fixed	1.14	0.89 to 1.47	.39	.13	.07	.30

Abbreviations

AF, atrial fibrillation; AFL, atrial flutter; AT, atrial tachycardia; CI, confidence interval; ETI, esophageal thermal injury; HPSD, high‐power short duration; LPLD, low‐power long duration; PE, pericardial effusion; PNP, phrenic nerve paralysis; PVI, pulmonary vein isolation; PVR, pulmonary vein reconnection; RR, risk ratio.

### 3.4 Outcomes

**FIGURE 2:**
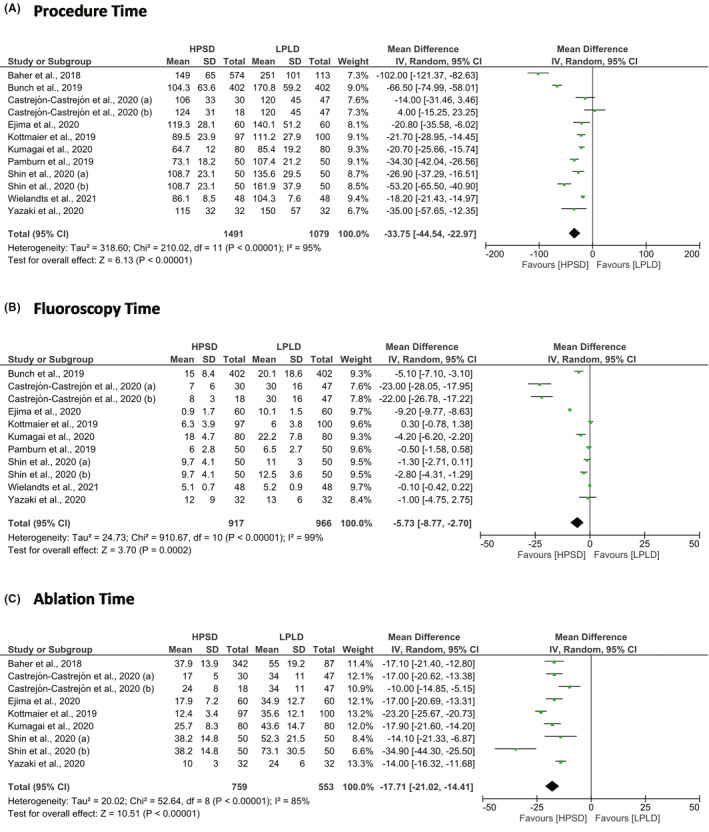
Forest plot of procedural parameters. (A) Procedure time; (B) Fluoroscopy time; and (C) Ablation time. CI, confidence interval; HPSD, high‐power short‐duration; IV, inverse variance; LPLD, low‐power long‐duration; SD, standard difference

**FIGURE 3:**
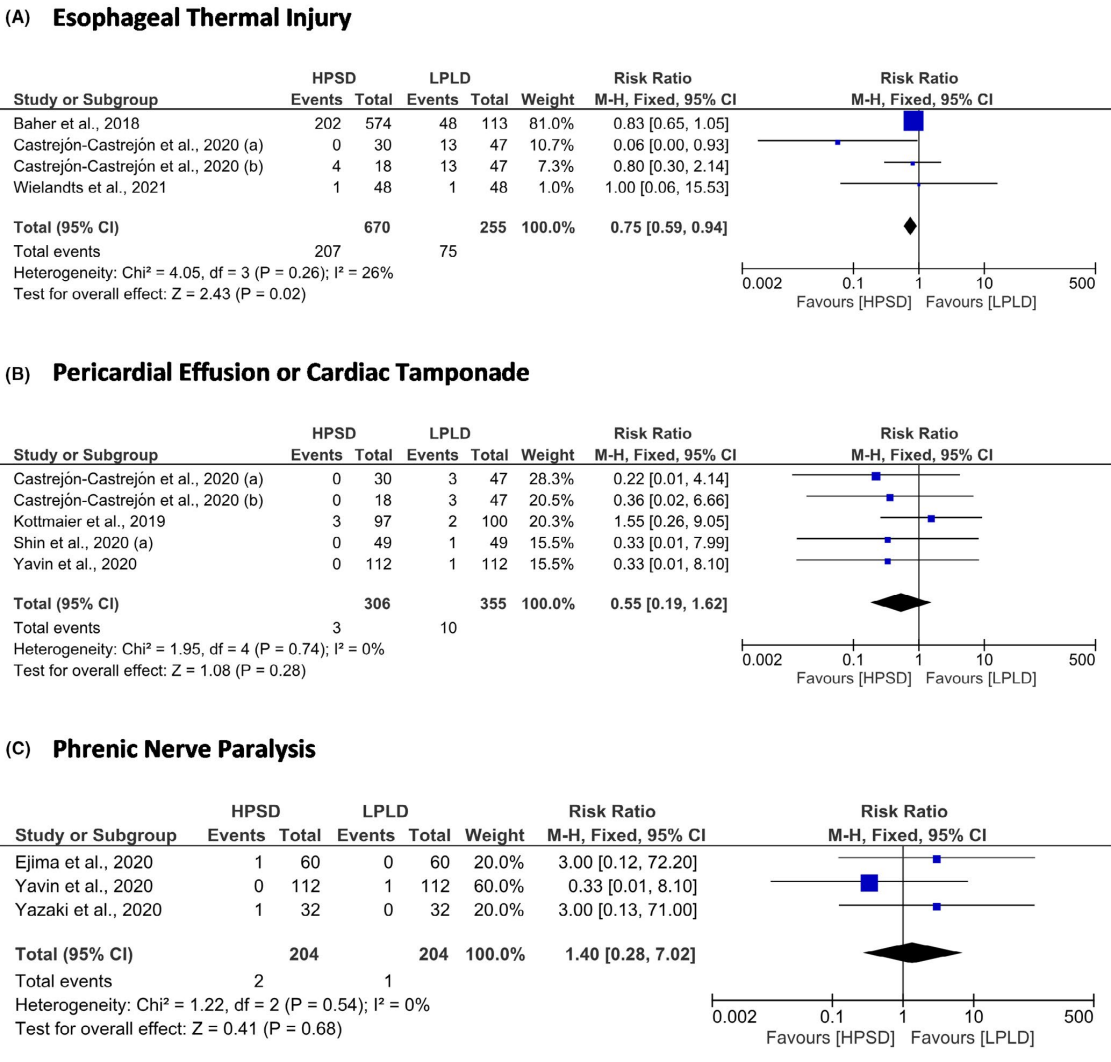
Forest plot of the safety outcomes. (A) Esophageal thermal injury; (B) Pericardial effusion or cardiac tamponade; and (C) Phrenic nerve paralysis. CI, confidence interval; HPSD, high‐power short‐duration; LPLD, low‐power long‐duration; M‐H, Mantel‐Haenszel

The HPSD ablation strategy took shorter the procedure time (MD = −33.75 min; 95% CI = −44.54 to −22.97; *P* < .01), fluoroscopy time (MD = −5.73 min; 95% CI = −8.77 to −2.70; *P* < .001), and ablation time (MD = −17.71; 95% CI = −21.02 to −14.41) than LPLD ablation strategy (Figure 2). From the safety aspects, the HPSD ablation strategy was associated with lower ETI than LPLD ablation strategy (RR = 0.75; 95% CI = 0.59 to 0.94; *P* = .02). However, the risk of pericardial effusion or cardiac tamponade (RR = 0.55; 95% CI = 0.19 to 1.62; *P* = .28) and phrenic nerve paralysis (RR = 1.40; 95% CI = 0.28 to 7.02; *P* = .68) in both groups were not significantly different (Figure 3).

**FIGURE 4:**
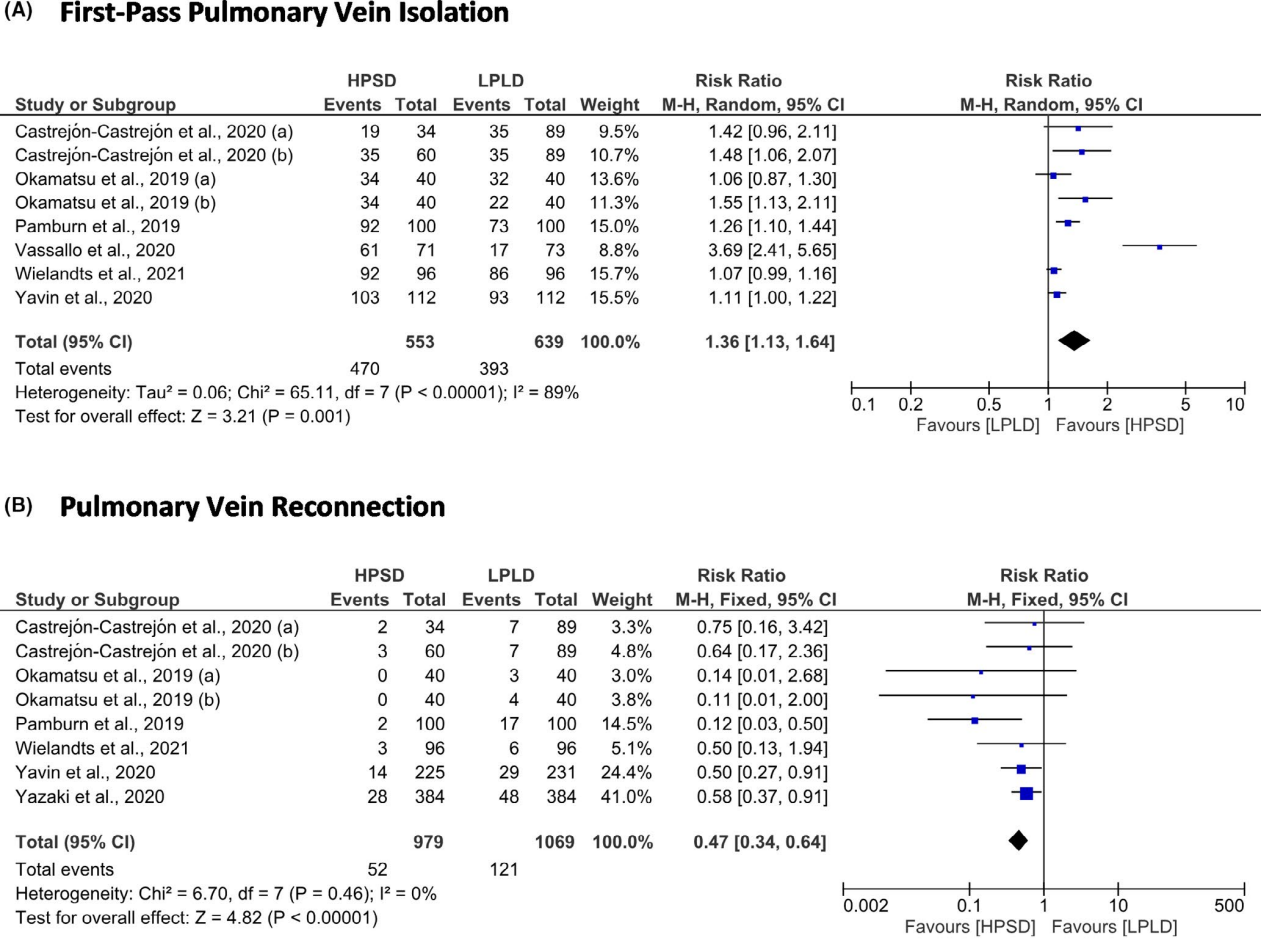
Forest plot of the short‐term efficacy outcomes. (A) First‐pass pulmonary vein isolation and (B) Pulmonary vein reconnection. CI, confidence interval; HPSD, high‐power short‐duration; LPLD, low‐power long‐duration; M‐H, Mantel‐Haenszel

**FIGURE 5:**
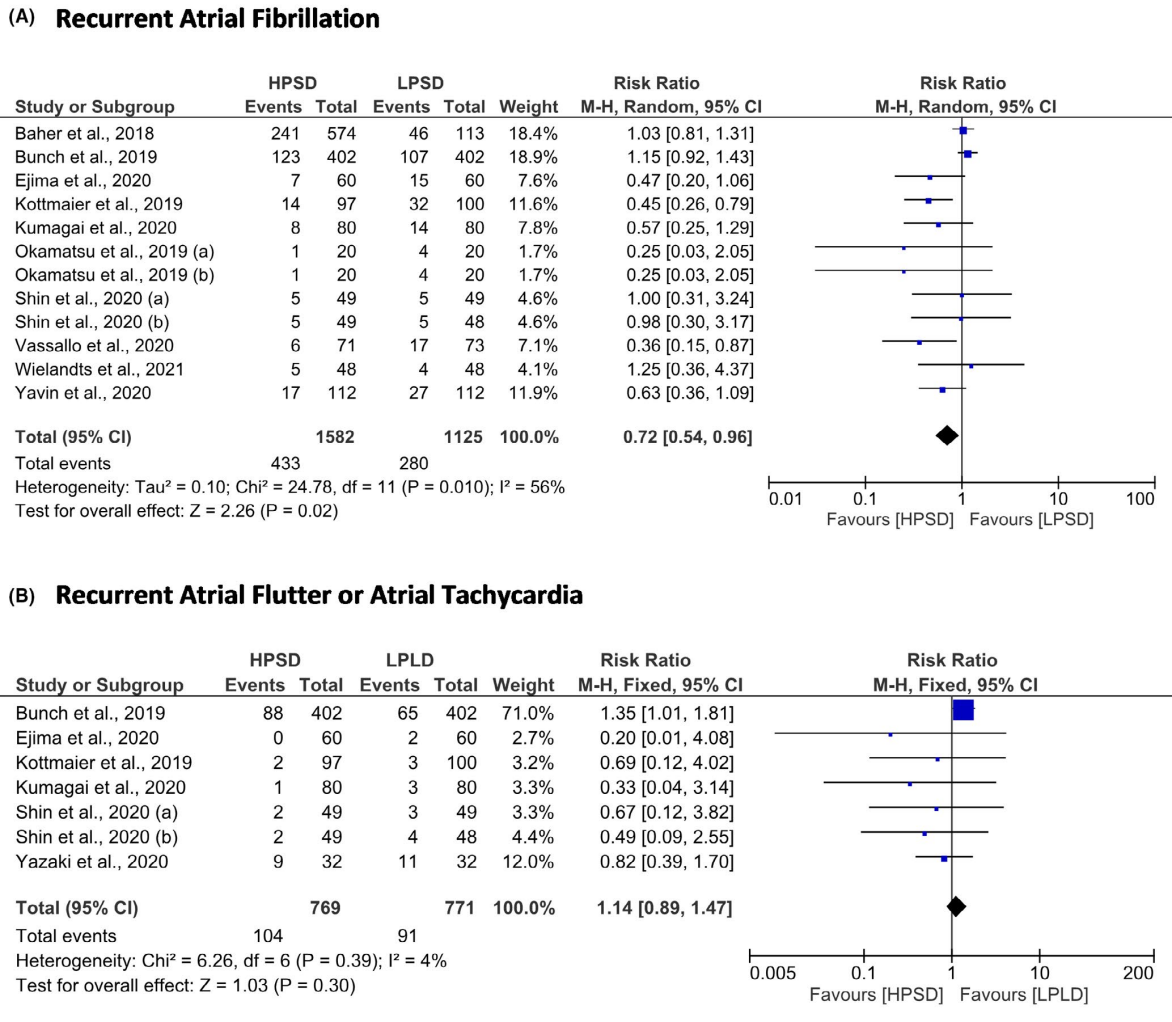
Forest plot of the long‐term efficacy outcomes. (A) Recurrent atrial fibrillation and (B) Recurrent atrial flutter or atrial tachycardia. CI, confidence interval; HPSD, high‐power short‐duration; LPLD, low‐power long‐duration; M‐H, Mantel‐Haenszel

We divided the efficacy outcomes into short‐term and long‐term efficacy outcomes. Short‐term efficacy outcomes included first‐pass PV isolation and PV reconnection. The HPSD ablation strategy was correlated with higher first‐pass PV isolation (RR = 1.36; 95% CI = 1.13 to 1.64; *P* < .01) and lower PV reconnection (RR = 0.47; 95% CI = 0.34 to 0.64; *P* < .01) than LPLD ablation strategy (Figure 4). The recurrent AF and recurrent AFL or AT were the long‐term efficacy outcomes. Conducting RFCA using HPSD approach significantly reduced the risk of recurrent AF (RR = 0.72; 95% CI = 0.54 to 0.96; *P* = .02). However, the risk of recurrent of AFL or AT was not significantly different in both groups (RR = 1.14; 95% CI = 0.89 to 1.47; *P* = .30) (Figure 5).

## 4 DISCUSSION

Several essential findings were obtained from this systematic review and meta‐analysis study. First, conducting AF catheter ablation using the HPSD approach was more efficient than the LPLD approach due to shorter procedure time, fluoroscopy time, and ablation time. Second, compared with LPLD RFCA, the HPSD approach reduced the risk of ETI. Third, HPSD was associated with greater first‐pass PVI. Fourth, the HPSD ablation method successfully reduced the risk of PV reconnection and recurrent AF following a single RFCA procedure.

### 4.1 The role of ablation power and duration

An effective and efficient PVI can be achieved by: (1) conduction block by transmural lesion generation; (2) sustained conduction block by cellular death and scar tissue formation; and (3) minimal cardiac injury.^13^, ^32^ RFCA aims to effectively convert electromagnetic energy into thermal energy to eradicate arrhythmogenic substrate in the myocardial tissue.^38^ Thermal injury caused by RFCA includes two sequential phases of resistive and conductive heating. The lesion generated by resistive or conductive heating depends on the balance between power and duration of RF application.^39^ During resistive heating, a resistive component located close to the tip of the catheter causes energy dissipation and local heating.^38^ Resistive heating achieves the maximum value within few seconds following RF energy delivery. During RFCA using the conventional LPLD method (power 25‐30 W), the temperature rises above 50°C. However, tissue necrosis occurs within a radius of 1‐1.5 mm from the tip of the ablation catheter.^40^ The higher power application can generate greater resistive heating. During conductive heating, the heat spreads to the deeper tissues passively. Conductive heating really depends on the RF application time. The longer RF application duration generates deeper and more extensive tissue heating.^5^, ^8^, ^9^, ^10^, ^11^ It also may increase the procedural complications associated with more extensive unnecessary tissue damage such as ETI, pericardial effusion, cardiac tamponade, or PNP.

The mean human left atrium thickness is 2.8 ± 1.1 mm and 1.7 ± 0.8 mm for superior and inferior levels, respectively.^41^ A study in AF patients undergoing PVI reported that based on the CT‐scan assessment, the mean thickness of the left atrial wall was 2.15 ± 0.47 mm, 1.43 ± 0.44 mm, and 1.81 ± 0.44 mm in the roof, posterior wall, and floor parts, respectively. The maximum left atrium thickness was 3.5 mm.^42^ The mean space between the left atrial posterior wall and the esophagus is 2.3 ± 1.2 mm.^43^ The RF energy application duration of 20‐30 seconds during the LPLD strategy depends on the earlier in vivo studies on ventricular tissue using non‐irrigated ablation catheters. Using a power of 25 W and a duration of 30 seconds, the RFCA generated the lesion with a mean depth of 7.25 mm.^8^ That LPLD ablation strategy could be appropriate for ventricular tachyarrhythmia ablation because the ventricular wall thickness is 3‐5 mm and 12‐15 mm for right and left ventricles, respectively.^44^, ^45^ However, the LPLD RF application could cause more extensive tissue lesions and collateral damage if applied in thin atrial tissue.

The HPSD strategy was developed to overcome the drawbacks of the conventional LPLD strategy. However, the HPSD approach had several possible limitations. First, the high power does not indicate the unlimited power rising.^46^ Second, the ideal power and duration limit of RF energy delivery is still unclear.^25^, ^26^, ^27^, ^28^, ^29^, ^30^, ^31^, ^32^, ^33^, ^34^, ^35^, ^36^, ^37^ Third, the efficacy and safety of the HPSD approach compared with the LPLD approach is still need to be confirmed. Those facts led us to perform this systematic review and meta‐analysis.

Several preclinical studies supported the benefit of HPSD RFCA for AF. An in silico study by Bourier et al. demonstrated that the HPSD ablation strategy generated wider and shallower lesions than the conventional LPLD approach.^13^ The experimental study in swine ventricles by Ali‐Ahmed et al. revealed that HPSD ablation using the power of 50 W, duration of 5 seconds, could create the lesion with the mean surface width, mean maximum width, and mean depth of 6.3 to 6.7 mm, 7.2 to 7.3 mm, and 2.9 to 3.0 mm, respectively.^10^ Leshem et al. also conducted an experimental study in swine hearts. Their study revealed that the HPSD method generated more extensive lesions with similar depth and greater lesion‐to‐lesion uniformity. In HPSD RFCA, the heat generated during the resistive phase can affect the tissue until the depth of 3.5 to 4 mm.^5^ It is suitable for atrial tissue because the maximum left atrial wall thickness is about 3.5 mm.^42^

### 4.2 Safety and efficacy outcomes

We included 13 studies in the meta‐analysis. The RFCA procedures in each study were conducted under the direction of the 3D electro‐anatomical mapping system.^25^, ^26^, ^27^, ^28^, ^29^, ^30^, ^31^, ^32^, ^33^, ^34^, ^35^, ^36^, ^37^ Most RFCA procedures were performed using the contact force‐sensing ablation catheter.^25^, ^26^, ^27^, ^28^, ^30^, ^31^, ^32^, ^33^, ^34^, ^35^, ^36^, ^37^ In the HPSD group, the maximum power ranged from 45 to 70 W. The power applied in the posterior wall was lower than the anterior wall. The radiofrequency application duration for each point in the LPLD group was <30 seconds. However, in the LPLD group, the maximum power used ranged from 30 to 40 W. The power applied in the posterior wall was also lower than the anterior wall. The radiofrequency application duration for each point in the HPSD group ranged from 3 to 42 seconds (Table 1).^25^, ^26^, ^27^, ^28^, ^29^, ^30^, ^31^, ^32^, ^33^, ^34^, ^35^, ^36^, ^37^ To simplify the data analysis process, HPSD was defined as the RFCA performed using the highest power of ≥40 W and the duration of ≤10 seconds in any ablation or less than duration in the LPLD group. On the other hand, LPLD was defined as the RFCA conducted using the highest power of <40 W and the duration of ≥10 seconds in any ablation or longer than duration in the HPSD group. As we expected, the HPSD ablation strategy effectively reduced the procedural time, fluoroscopy time, and ablation time. It was supported the results from prior meta‐analysis studies.^12^, ^46^, ^47^, ^48^

From the safety aspects, our meta‐analysis revealed that performing RFCA for AF using the HPSD approach could reduce the incidence of ETI. It was not similar to the results of the previous meta‐analysis studies.^46^, ^47^ We added the results of the study from Wielandts et al.^35^ and analyzed the results of the study from Castrejón‐Castrejón et al.^27^ using a different approach from the prior meta‐analysis from Chen et al.^46^ and Li et al.^47^ In our study, we divided the HPSD group (the study from Castrejón‐Castrejón et al.^27^) into 50 W and 60 W groups to be involved in the statistical analysis. The reduction of ETI risk using the HPSD approach was found in the study from Castrejón‐Castrejón et al.^27^ using the power of 60 W. On the other hand, the results from the other studies (Baher et al.^25^ [power of 50 W], Castrejón‐Castrejón et al.^27^ [power of 50 W], and Wielandts et al.^35^ [power of 50 W]) failed to prove the net benefit of ETI risk reduction using HPSD RFCA approach (Figure 3A). It was suggested that the advantage of the ETI risk reduction of the HPSD approach was more significant using the higher power. In the study from Castrejón‐Castrejón et al.,^27^ the HPSD RFCA using the power of 60 W took shorter radiofrequency application time than HPSD RFCA using the power of 50 W (17 ± 5 min vs 24 ± 8 min; *P* < .01).^27^ It seemed that the risk of ETI was more associated with the duration of RFCA, not the power.

Both groups did not show a significant difference for PNP, pericardial effusion, and cardiac tamponade. Those data were not reported in several prior meta‐analysis studies.^12^, ^46^, ^47^ Based on the basic principle of HPSD increases resistive heating and reduces conductive heating, minimizing collateral tissue damage is the advantage of HPSD RFCA. Our systematic review and meta‐analysis revealed that the HPSD approach was a safe procedure. Several studies in animals and humans had confirmed the lower complication rate of the HPSD approach.^5^, ^9^, ^25^, ^49^

For the short‐term efficacy outcomes, our study revealed that HPSD RFCA had better first‐pass PVI than LPLD RFCA. This result was consistent with the findings from the previous meta‐analysis.^46^, ^47^, ^48^ Moreover, the HPSD approach was also associated with a lower risk of PV reconnection. The data about the risk of PV reconnection were provided by the meta‐analysis study from Ravi et al.^48^ and our results supported that. For the long‐term efficacy outcomes, our study demonstrated that performing HPSD RFCA effectively reduced the risk of recurrent AF. However, the HPSD method failed to reduce the risk of recurrent AFL or AT. The data about recurrent AF and recurrent AFL or AT were not reported in prior meta‐analysis studies,^12^, ^46^, ^47^, ^48^ and our meta‐analysis study provided data about that. Those prior meta‐analysis studies used freedom from atrial tachyarrhythmias as the efficacy endpoint.^12^, ^46^, ^47^, ^48^

The ability of the HPSD method in increasing first‐pass PVI and reducing PV reconnection and recurrent AF was due to larger area, more uniform, and more consistent lesion generated by the HPSD method. The stability of catheter‐tissue contact is the essential part contributing to lesion formation. The instability of the ablation catheter in the beating heart can disrupt the RF energy delivery from the catheter to the tissue.^32^ The reduction of ablation time can minimize catheter instability and perhaps improve lesion generation by increasing the possibility of maintaining catheter stability during the RF energy application.^50^ The catheter instability was a problem in the LPLD approach that leads to various lesions, tissue edema, wider tissue damage, lower rate first‐pass PVI, and higher rate of PV reconnection. The perfect PVI with transmural scar formation is an essential factor in ensuring freedom from recurrent AF.^51^, ^52^ Therefore, the HPSD RFCA can provide permanent lesions with better continuity and transmurality. Theoretically, the relatively shorter RF application duration and consequently smaller RF energy delivery in the LPLD RFCA can reduce the first‐pass PVI, increase the PV reconnection, and increased AF recurrence. However, to the best of our knowledge, no specific study directly compared long duration versus short duration of RF energy application using similar low power (<40 W) in both groups. Moreover, the research that compares high power versus low power RFCA using equal duration is still not available. Our study also showed that the HPSD method could not reduce the risk of recurrent AFL or AT. This result could be caused by several factors: (i) different arrhythmogenic mechanisms, (ii) some patients received not only PVI, and (iii) AFL or AT could be caused by scar formation due to RFCA.^51^, ^53^

### 4.3 Study limitations

We recognized several limitations in this systematic review and meta‐analysis. First, most studies involved in this study were cohort studies, causing unwanted selection bias and referral bias. However, we overcame this situation by including only high‐quality studies. Second, the publication bias possibility could not be avoided. To minimize this situation, we performed a double publication bias evaluation using Begg’s and Egger’s tests. We did not find any publication bias in this meta‐analysis. Third, the settings of power and duration in the involved studies were not uniform. Fourth, the variation in the follow‐up period duration and arrhythmia detection methods could be confounding factors. Fifth, the inability to get data at individual patient‐level limited our effort to determine the real effects at the patient level.

## 5 CONCLUSION

Our systematic review and meta‐analysis study revealed that the HPSD RFCA was a safe, effective, and efficient procedure to treat AF. The superiority offered by the HPSD method over the conventional LPLD strategy method: (i) shorter procedure time, fluoroscopy time, and ablation time; (ii) lower risk of ETI; (iii) higher first‐pass PVI; and (iv) lower risk of PV reconnection and recurrent AF. However, the universal definition of HPSD RFCA was not available. Our findings suggested that the RCT with a large number of patients, better design, more clear HPSD definition, longer follow‐up duration, and more appropriate arrhythmia detection methods should be conducted.

## CONFLICT OF INTEREST

All authors declare no conflict of interest regarding the publication of this article.

## Supporting information

Table S1‐2
