# Supplementary material for: The superiority of high‐power short‐duration radiofrequency catheter ablation strategy for atrial fibrillation treatment: A systematic review and meta‐analysis study
Source: J Arrhythm. 2021 Jul 2;37(4):975–89. doi: 10.1002/joa3.12590 (PMC8339091; doi:10.1002/joa3.12590)
Supplement: Supplementary file 1 — Table S1‐2 [file JOA3-37-975-s001.docx]

Table S1. Newcastle-Ottawa Scale for cohort studies.

| Study | Selection | | | | Comparability | Outcome | | | NOS | Quality |
| --- | --- | --- | --- | --- | --- | --- | --- | --- | --- | --- |
|  | Representativeness of the exposed cohort | Selection of the non-exposed cohort | Ascertainment of exposure | Demonstration that outcome of interest was not present at the start of the study | Comparability of cohorts based on the design or analysis controlled for confounders | Assessment of outcome | Was follow-up long enough for outcomes to occur | Adequacy of follow-up of cohorts |  |  |
| Baher et al., 2018 | 1 | 1 | 1 | 1 | 1 | 1 | 1 | 1 | 8 | Good |
| Bunch et al., 2019 | 1 | 1 | 1 | 1 | 1 | 1 | 1 | 1 | 8 | Good |
| Castrejón et al., 2020 | 1 | 1 | 1 | 1 | 1 | 1 | 1 | 1 | 8 | Good |
| Ejima et al., 2020 | 1 | 1 | 1 | 1 | 2 | 1 | 1 | 1 | 9 | Good |
| Kottmaier et al., 2019 | 1 | 1 | 1 | 1 | 2 | 1 | 1 | 1 | 9 | Good |
| Kumagai et al., 2020 | 1 | 1 | 1 | 1 | 2 | 1 | 1 | 1 | 9 | Good |
| Okamatsu et al., 2019 | 1 | 1 | 1 | 1 | 2 | 1 | 1 | 1 | 9 | Good |
| Pamburn et al., 2019 | 1 | 1 | 1 | 1 | 2 | 1 | 1 | 1 | 9 | Good |
| Vassallo et al., 2020 | 1 | 1 | 1 | 1 | 2 | 1 | 1 | 1 | 9 | Good |
| Yavin et al., 2020 | 1 | 1 | 1 | 1 | 1 | 1 | 1 | 1 | 8 | Good |
| Yazaki et al., 2020 | 1 | 1 | 1 | 1 | 1 | 1 | 1 | 1 | 8 | Good |

NOS = Newcastle-Ottawa scale.

Table S2. Modified Jadad scale for randomized controlled trials.

| Study | Was the study described as randomized? | Was the method of randomization appropriate? | Was the study described as blinding? | Was the method of blinding appropriate? | Was there a description of withdrawals and dropouts? | Was there a clear description of the inclusion/exclusion criteria? | Was the method used to assess adverse effects described? | Were the methods of statistical analysis described? | Modified Jadad Scale | Quality |
| --- | --- | --- | --- | --- | --- | --- | --- | --- | --- | --- |
| Shin et al., 2020 | 1 | 1 | 0.5 | 0 | 1 | 1 | 1 | 1 | 6.5 | Good |
| Wielandts et al., 2021 | 1 | 1 | 0 | 0 | 0 | 1 | 1 | 1 | 5 | Good |
